# Comparison of Grip Strength, Forearm Muscle Activity, and Shock Transmission between the Forehand Stroke Technique of Experienced and Recreational Tennis Players Using a Novel Wearable Device

**DOI:** 10.3390/s23115146

**Published:** 2023-05-28

**Authors:** Chantelle Jean Rigozzi, Gareth A. Vio, Philip Poronnik

**Affiliations:** 1 FMH Media Lab, School of Medical Sciences, Faculty of Medicine and Health Sciences, The University of Sydney, Sydney 2006, Australia; philip.poronnik@sydney.edu.au; 2 School of Aerospace, Mechanical and Mechatronic Engineering, The University of Sydney, Sydney 2006, Australia; gareth.vio@sydney.edu.au

**Keywords:** elbow tendinopathy, wearable technology, sport biomechanics, electromyography, IMU, accelerometer, gyroscope, statistical parametric mapping

## Abstract

Upper limb tennis injuries are primarily chronic, resulting from repetitive overuse. We developed a wearable device which simultaneously measures risk factors (grip strength, forearm muscle activity, and vibrational data) associated with elbow tendinopathy development resulting from tennis players’ technique. We tested the device on experienced (*n* = 18) and recreational (*n* = 22) tennis players hitting forehand cross-court at both flat and topspin spin levels under realistic playing conditions. Using statistical parametric mapping analysis, our results showed that all players showed a similar level of grip strength at impact, regardless of spin level, and the grip strength at impact did not influence the percentage of impact shock transfer to the wrist and elbow. Experienced players hitting with topspin exhibited the highest ball spin rotation, low-to-high swing path brushing action, and shock transfer to the wrist and elbow compared to the results obtained while hitting the ball flat, or when compared to the results obtained from recreational players. Recreational players exhibited significantly higher extensor activity during most of the follow through phase compared to the experienced players for both spin levels, potentially putting them at greater risk for developing lateral elbow tendinopathy. We successfully demonstrated that wearable technologies can be used to measure risk factors associated with elbow injury development in tennis players under realistic playing conditions.

## 1. Introduction

The forehand stroke is the most used groundstroke in professional tennis matches [1], and it is often the first stroke taught to recreational tennis players [2]. Muscle tendon injuries are the most common type of injury seen in elite tennis players, with overuse injuries more prevalent in the upper extremity [3]. Improper technique and different forehand grip positions have been suggested as risk factors for the development of elbow and wrist tendinopathy in tennis players [4,5,6]. Additionally, the player’s grip strength, forearm muscle activity, and the vibrational transfer up the forearm after impact have been suggested as risk factors due to the repetitive nature of the strokes and the frequency of post-impact forces [4,7,8].

The rapid and ongoing development of wearable technologies allows for the kinematic parameters associated with tennis player technique to be measured under realistic playing conditions [9,10,11,12]. In the past, biomechanics studies on the kinematic parameters of tennis player technique have relied on the use of laboratory confined motion capture systems (e.g., Kwon, Pfister [13], Loushin, Kakar [14], Ikenaga, Okuma [15]). Although optical motion capture systems are widely accepted as the gold-standard for motion capture in sports biomechanics, due to their small size and portability, the use of wearable devices for player motion analysis is becoming an increasingly popular method to measure the kinematic parameters associated with player ball hitting technique in tennis [16].

The most common types of sensors used in these wearable devices are inertial measurement unit sensors (IMU) and electromyography sensors [16]. IMU sensors use a combination of accelerometers (acceleration), gyroscopes (angular velocity), and magnetometer sensors (magnetic forces) [17,18] and can be used for kinematic motion analysis in tennis [19]. They can measure the player’s racket face angle (gyroscope), racket head acceleration (accelerometer), and vibrational transfer (multiple accelerometers). Data from these sensors can either be analyzed separately or integrated using fusion algorithms, and the sensors have been proven to have a level of accuracy comparable to that of optical motion capture systems used in sports biomechanics [20,21,22,23]. Surface electromyography sensors measure the muscle electromyography (EMG) activity and can be used to relate muscle force output over a period of time [24]. Additionally, pressure sensors can measure changes in the player’s grip force over the stroke [25,26].

Special commercialized tennis racket devices, such as Babolat Play^®^ and the Head Tennis Sensor^®^, have been developed to help players understand their overall game in more detail through the use of algorithms to estimate the type of swing played, number of strokes hit, impact location of the ball on the racket face, ball exit spin level, and ball exit speed under playing conditions. However, the accuracy of these commercially available tennis sensors is currently unknown due to the fact that the technical specifications of the sensors, the software, and the analysis methods are not publicly available; there is also no requirement for these sensors to conform to the technical quality control of data standards [27]. We predict that the ability to easily visualize and analyze the players’ technique using wearable technology could improve their technique and potentially reduce their risk of developing upper extremity injuries.

### 1.1. The Forehand Stroke, Spin Levels, and Grip Positions

The forehand stroke is a complex series of movements, and it is often unique to each player [28]. The forehand stroke can be divided into three phases: preparation, acceleration, and follow-through. The preparation phase is characterized by the first movement of the backswing to the first forward movement of the racket, the acceleration phase is characterized by the first forward movement of the racket to the point of ball impact, and the follow-through phase starts at the point of impact and finishes with completion of the stroke [29]. The racket face angle and racket swing path dictate the accuracy of the shot, while the ball exit speed is primarily governed by the velocity of the incoming ball and the racket head speed (combination of the racket horizonal, lateral and vertical velocities) [30]. Faster racket head velocity results in greater ball exit speed during the forehand stroke [31]. A study by Knudson and Blackwell [32] found that during the topspin forehand stroke, high-performance players were able to hit the forehand stroke with higher ball exit speed and more low-to-high swing path action as the racket approached impact, as well as with a more closed racket face angle compared with intermediate-level players. Functional variability allows for increased variation in players’ technique to compensate for differences in other aspects of their stroke to ensure consistent shot outcomes [33,34].

The forehand stroke is often hit with varying levels of spin, racket head speed, and grip positions, depending on the player’s playing ability and preference. The two main spin levels in the forehand stroke are flat (minimal ball rotation) and topspin (moderate level of forward rotation) [35]. Although topspin is the most effective method for controlling the ball on the court, recreational players will typically first learn to hit the ball flat before they learn topspin [36,37]. Topspin is generated on the ball by the racket following a low-to-high swing path (wrist abduction) through the impact zone [31]. The racket face angle is either vertical or nearly vertical at impact when the ball is hit with either flat [31] or topspin [32] spin levels. Kinematic studies have shown a decrease in horizontal and an increase in vertical racket head velocity between the flat and topspin forehand strokes [35,37,38]. Interestingly, this movement of wrist abduction involves both the extensor carpi radialis (ECR) and flexor carpi radialis (FCR) muscles [39]. Although previous studies have found that hitting the ball flat resulted in faster ball exit speeds than hitting the ball with topspin [10,37,40], a study by Rota, Hautier [41] found no relationship between ball exit speed and the activation level of the ECR and FCR muscles.

The way in which the player holds the racket is one of the most important aspects of stroke mechanics [30]. The different forehand grip positions influence the alignment of the wrist at impact and the kinematics of the forehand stroke [42]. Typically, the player’s grip position arises from the coaching environment in which they have learned the game and the spin and height they prefer for hitting the ball [43]. There are four traditional single-handed forehand grip positions (Continental, Eastern, semi-Western, and Western) [6,43]. Players with a Western grip use more palmar flexion to help generate vertical racket head velocity, while those with an Eastern grip use more radial flexion [35,44]. Compared to the results of earlier studies, wrist injuries are found to occur more frequently in the modern game where more topspin is often hit [45], with a study by Tagliafico, Ameri [6] showing that ulnar-sided wrist injuries were associated with the semi-Western or Western grip, while radial-sided injuries were associated with the Eastern grip.

### 1.2. Elbow Tendinopathy

It has been previously reported that between 40–50% of players experience elbow injury symptoms throughout their tennis careers [46,47]. Lateral elbow tendinopathy (LET) or “tennis elbow” is more common in recreational players and is typically associated with overuse of the extensor carpi radialis brevis (ECRB) muscle [48], while medial elbow tendinopathy (MET), or “golfer’s elbow”, is more common in advanced players [49,50] and is commonly associated with overuse of the FCR muscle [4]. The repetitive overload on the forearm musculature during the forehand stroke [4,51], along with vibration frequencies in the range of 80–200 Hz [8], have been suggested as underlying causes of the development of elbow tendinopathy in tennis players. The forehand stroke is thought to play a main role in the development of MET, especially among tennis players who often hit the ball with topspin, due to valgus overload during the acceleration phase [4]. To date, there is no consensus in the literature as to the main etiology of elbow tendinopathy, although it has been suggested to be multifactorial [52] and non-gender specific [53]. One commonly accepted theory to describe the development of elbow tendinopathy in tennis players is based on the stiffness of the skeletal muscle units and their repetitive overuse in tennis strokes [10]. The vibrations from impact are transferred efficiently and directly to the myotendinous junction, causing repeated microtrauma when the skeletal muscles stiffen near maximum contraction [8,54]. However, there is no clinical evidence to demonstrate a link between vibration and elbow tendinopathy [55].

### 1.3. Grip Stength, Racket Vibration, and Impact Location

Post-impact forces transmitted to the hand and arm during the forehand stroke are a complex integration of grip strength, racket vibration, and impact location [56]. In the past, tennis coaches instructed beginners to use a firm grip while hitting the forehand stroke [57]. However, most players will naturally learn to use enough grip force to control the racket during the forehand stroke and only increase it just prior to impact [44]. The mechanical coupling of the racket handle with the player’s body changes with each modification of grip force due to the forearm muscles continuously modifying the grip forces on the racket handle [58,59]. Although the ball exit speed is not influenced by the magnitude of grip force [57,60], higher grip force and ball exit speed increase the transfer of shock and vibration to the player’s arm [57,59,61,62].

The location where the ball strikes the racket face is one of the most important factors in determining the post impact peak forces transmitted to the hand, as well as the subsequent frame vibrations [27]. The frequency of the frame vibrations depends on the time it takes for the initial bending wave to travel along the frame from the impact point location, bounce off the ends, and return back to the starting point [63]. There is a curved node line extending from the middle of the strings to points at about 2 and 10 o’clock on the frame, and if the ball hits anywhere along this curved line, the racket will not vibrate [63]. The commonly referred to “sweet spot” is the intersection of this curved line with the long axis of the racket [63]. It is often thought that experienced players hit the ball more consistently in the sweet spot area compared with less proficient players [8].

### 1.4. Previous Relevant Research

Previous forehand tennis studies have used IMUs to analyze the contribution of different upper limb segments to racket head speed [64] and rotation of the waist during the stroke [12]. Another tennis study used surface electromyography and tri-axial accelerometers to measure forearm muscle activity and vibrational transfer from the racket to the hand and forearm during the forehand stroke under realistic playing conditions [65]. However, the study by Yeh, Elangovan [65] required players to play until near exhaustion while investigating the influence of a racket with a novel vibration damping technology and did not measure the player’s racket face angle, grip strength, ball spin level, or average ball exit speed. Other previous studies by Chadefaux, Rao [66], and Wei, Chiang [62] have investigated the player’s grip strength, forearm muscle activity, and vibrational transfer during the forehand and backhand stroke, respectively. However, these studies simulated the player’s grip strength using the forearm electromyography (EMG) activity, and ball impact location was not measured. Differences in grip strength and wrist vibration between expert and casual players has previously been investigated in a study by Schnabel and Hennig [67]. However, this study only measured vibrations at the wrist and did not measure forearm muscle activity. It should also be noted that previous relevant review articles have focused on the biomechanics, grip style, and pressure during the forehand stroke [42], as well as the use of wearable technology for player motion analysis in racket sports under playing conditions [16].

### 1.5. Aims and Hypothesis

This study is based on the hypothesis that the spin level, different levels of playing experience, and preferred forehand grip position will influence racket face angle, racket head acceleration, grip strength, forearm extensor and flexor activity, and vibrational transfer. This in turn may determine an individual’s risk of injury. We developed and tested a novel microcontroller-based wearable device that could simultaneously measure tennis player racket face angle, racket head acceleration, grip strength, forearm EMG activity, and vibrational data under realistic playing conditions. We were able to perform measurements on individuals with different levels of playing experience and forehand grip positions while they were hitting forehand shots of different spin levels. This allowed us to analyze the patterns of movement and compare the racket face angle, racket head acceleration, grip strength, forearm extensor and flexor activity, and vibrational transfer in each player.

## 2. Materials and Methods

### 2.1. Design of Device

**Figure 1:**
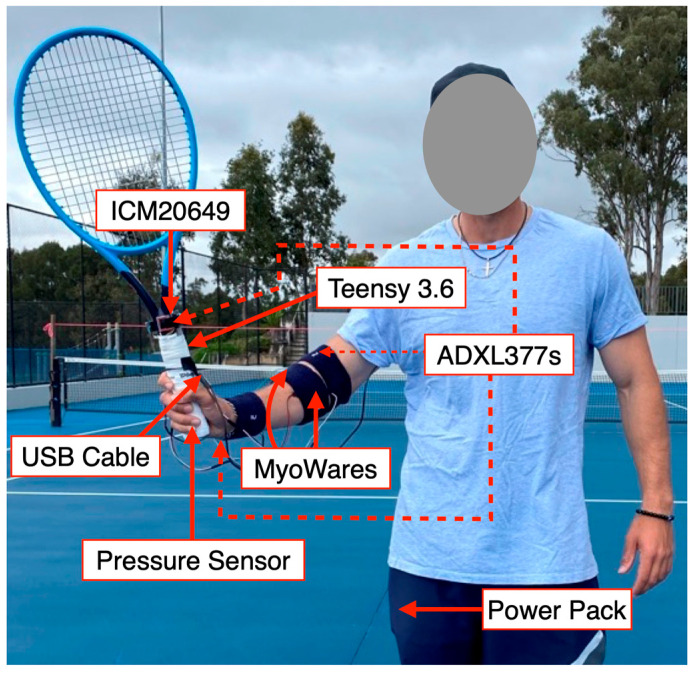
The placement of the Tennis Racket Accelerometer MyoWare Wearable Device Version 2 (TRAM-2) on a player is shown. TRAM-2 sampled all sensors simultaneously at 9000 samples per second. All TRAM-2 measurements were recorded relative to the racket (for sensors located on the racket) or the player’s arm (for sensors located on the player’s arm). The ICM20649 (accelerometer and gyroscope) and racket ADXL377 (accelerometer) *x*-axis was the direction across the string bed, the *y*-axis was the direction along the racket from the tip to the handle, and the *z*-axis was perpendicular to the string bed.

We designed, developed, and tested a prototype device called the Tennis Racket Accelerometer MyoWare Wearable Device Version 2 (TRAM-2). The design of the prototype device (TRAM-2) used has been reported in our previous study [9]. TRAM-2 is the first device that can simultaneously measure the players racket face angle, racket head acceleration, grip strength, forearm muscle activity, and vibrational data under realistic playing conditions. TRAM-2 consists of a microcontroller (Teensy 3.6, PJRC, Sherwood, OR, USA) attached to the handle of the racket (Head Graphene 360 Instinct MP, 4 3/8 inches, 300 g); a 6-DOF IMU (ICM20649 (±30 g-force (g), ±4000 degrees per second (dps)), Adafruit, New York, NY, USA) attached to the throat of the tennis racket; three 3-axis accelerometers (ADXL377 (±200 g), SparkFun, Boulder, CO, USA) attached to the racket, wrist (lateral epicondyle of the distal ulnar head), and elbow (lateral epicondyle of the humerus) using double sided tape; a custom-built pressure sensor (7 sheets of Adafruit Velostat between two sheets of Adafruit Copper Foil Sheet), located around the racket handle; and two EMG Muscle Sensors (MyoWare^®^ AT-04-001, Advancer Technologies, Raleigh, NC, USA), which are positioned over the main forearm muscles associated with the development of elbow tendinopathy (ECRB and FCR) (Figure 1). The sampling rate of the ICM20649 (9000 samples per second) was used to trigger the collection of all other sensor data simultaneously, enabling each data point from all the sensors to have the same timestamp. Using a modified version of an open access low latency data-logging Teensy 3.6 SDIO SD code, the collected data were stored on a micro-SD card (SanDisk Ultra microSDHD UHS-I 16 GB Card) on the Teensy [68]. All TRAM-2 measurements were recorded relative to the either the racket (for sensors located on the racket) or the player’s arm (for the sensors located on the players arm). Two TRAM-2 rackets (average weight 376 ± 1 g) were identically prototyped for this experiment. Each player used the same racket throughout the whole testing session, and the TRAM-2′s recording functionality and appropriate MyoWare^®^ placement were verified on each player using guided wrist extensor and flexor manual muscle tests.

The original tennis racket grip was removed from the racket handle and replaced with the custom-built pressure sensor. The pressure sensor was then wrapped with two white Wilson Pro Overgrips^®^. The device was powered by a portable power bank (Cygnett, 3000 mAh) in the player’s pocket, connected using a 1.2 m Micro-USB cable (Belkin Micro-USB) taped onto the player’s arm (Figure 1). The two MyoWares^®^ shared a reference electrode placed on the medial epicondyle of the humerus. As we have previously reported, due to the accuracy of surface EMG and the MyoWare^®^ sensors, we accept that our measurements were detecting components of all forearm extensor (ECR) and flexor (FCR) activity when reporting the ECRB and FCR MyoWare^®^ data, respectively [10]. Using sweatbands, the MyoWares^®^ and accelerometers were further secured to the player’s body.

### 2.2. Testing of the Device

Players who volunteered for the study were confident that they could successfully hit both flat and topspin spin levels. The device was tested on 40 tennis players. For statistical analysis, players were categorized based on their level of playing experience into either the experienced (must have either been ranked internationally, within the top 200 nationally, or have obtained a professional coaching qualification) or recreational (played tennis non-competitively at least once a week for the past 6 months) group. The physical characteristics of the 18 players categorized as experienced were males = 16, females = 2, age = 32.9 ± 10.6, height = 180.6 ± 5.7 cm, weight 81.6 ± 9.4 kg, and years of experience playing tennis = 24.7 ± 10.9. The 22 players categorized as recreational were males = 20, females = 2, age = 36.4 ± 12.8, height = 178.5 ± 8.1 cm, weight 78.2 ± 10.5 kg, and years of experience playing tennis = 19.1 ± 11.6. Overall, 37 players were right-handed and 3 players were left-handed. Due to the low number of female participants, additional gender comparisons were not performed.

**Figure 2:**
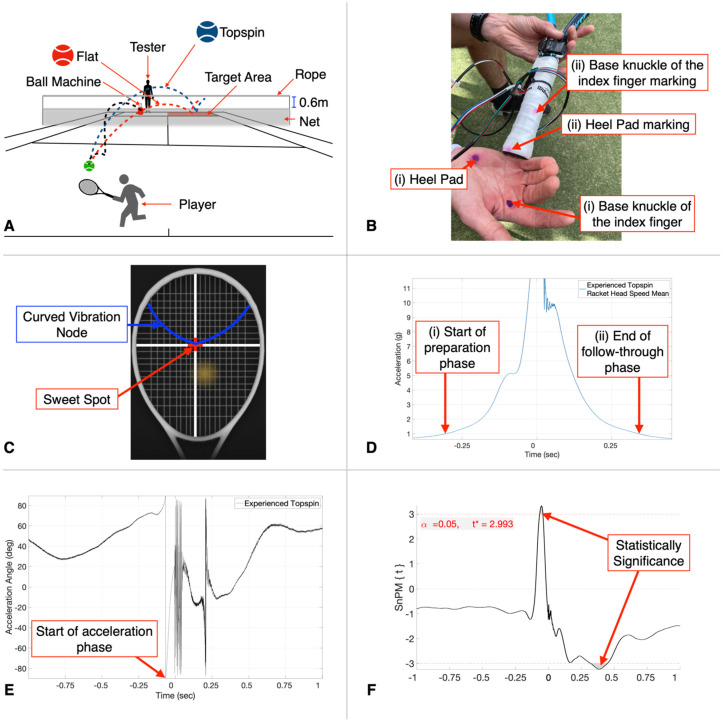
This figure shows experimental design and an output example of the continuous time series statistical non-parametric mapping analysis performed. (**A**) Experimental on-court testing protocol. For the shot to be include for analysis, players must have hit the ball from the ball machine into the target area, following the correct ball spin height guideline as indicated for either the flat (red dashed line) or topspin (blue dashed line) spin level. (**B**) Preferred forehand grip position methodology. The preferred forehand grip position of the player was determined by marking the player’s dominant hand in two locations ((i) base knuckle of the index finger and heel pad) prior to the commencement of testing. The players forehand grip position was determined depending on the racket bevel number associated with the mark observed after testing (ii). (**C**) Sweet spot accuracy. Location of the sweet spot (red) and curved vibrational node (blue) on the racket face. Using the Head Tennis Sensor App, if the mark representing the ball touched the curved vibrational node, the shot was marked as having correct sweet spot accuracy. (**D**) Timepoints associated with the start of the preparation phase (i) and end of the follow-through phase (ii); the racket head acceleration (vector sum of the racket ADXL377 3-axes) first reached above and below the threshold of 1 g before and after impact, respectively. (**E**) The inverse tangent of the racket ADXL377 *x*-axis over the *z*-axis was used to determine the timepoint associated with the start of the acceleration phase when −90° was first reached, before impact. (**F**) Statistical non-parametric mapping (SnPM) analysis representing a typical statistical non-parametric mapping output. Shaded regions represent statistical significance.

For the on-court testing session, a guided 5 min warmup consisting of hitting forehand shots cross-court at different spin levels (flat and topspin) into the target zone (back diagonal square) was required for all players (Figure 2A). The flat spin level was defined as the ball travelling under a rope placed 0.6 m above the height of the net, while the topspin spin level was defined as the ball travelling over this rope (Figure 2A). For the testing phase, each player was instructed to hit 40 forehand strokes cross-court, following the height guideline, into the target zone for each forehand spin level (flat and topspin) (Figure 2A). Hits were only included for analysis if all relevant sensors recorded successfully, and the ball followed the height guideline and landed in target area (determined by the same tester for all players).

New tennis balls (Wilson Tour Premier) were ejected from a ball machine (Lobster Elite Three, Lobster Sports, North Hollywood, CA, USA) (80 kph), with the timing set to 8 s. The tester was standing next to the ball machine, which was located 2 m towards the center mark from the singles line on the baseline opposite to the participant (Figure 2A). Players were required to perform 10 air swings (swings mimicking the forehand technique without hitting the ball) as a baseline control before the testing phase for comparison with the data when the ball was actually hit. Each player had 15 s rest between each series and a 5 min rest between each spin level. The spin level was randomized between players, and a GoPro Hero-10 (240 fps) video camera was stationed behind the player for additional reference.

Players were also categorized for statistical analysis based on their preferred forehand grip position. The player’s forehand grip position was determined by marking their dominant hand in two positions (base knuckle of the index finger and the heel pad of the palm) (Figure 2B(i)) [6,43] and observing where they held the racket during the forehand stroke. Depending on which racket handle bevel number the marks rubbed off onto during testing (Figure 2B(ii)), the player’s grip position was determined [6,10]. If both marks were not available (due to the players holding the racket lower on the handle), the base knuckle position alone was used to determine the forehand grip position. Of the 40 players, 21 players preferred the Eastern (experienced = 11, recreational = 10, age = 38.1 ± 13.5, height = 179.2 ± 6.5 cm, weight 80.5 ± 9.2 kg, and years of experience playing tennis = 24.6 ± 13.3), 17 players preferred the semi-Western (experienced = 7, recreational = 10, age = 30.9 ± 9.2, height = 180.0 ± 8.2 cm, weight 79.0 ± 11.7 kg, and years of experience playing tennis = 18.6 ± 8.5), and 2 players preferred the Continental (age = 33.5 ± 0.7, height = 177 ± 4.2 cm, weight 77.5 ± 0.7 kg, and years of experience playing tennis = 16 ± 5.7) forehand grip position. Due to the low number of players preferring the Continental grip position, only the Eastern and the semi-Western grip position groups were compared during statistical analysis. Ethical approval was obtained from the University of Sydney (Protocol 2019/434). No players reported any injury in their dominant upper limb in the last 6 months, and all were over 18 years of age.

### 2.3. Ball Exit Speed, Ball Spin Level, and Sweet Spot Accuracy

A Head Tennis Sensor^®^ replaced the butt cap of the tennis racket and measured the ball exit speed (kph), ball spin rotation (rotation per minute (rpm)), and impact location of the ball on the racket face corresponding to each hit through the accompanying mobile phone application (Table 1). The ball exit speed and spin rotation were noted for each correctly hit shot. To determine the level of sweet spot accuracy for each hit, a screen shot of the mobile phone application was captured, and the curved node line was drawn onto each racket face (Figure 2C). If the mark representing the ball touched the curved node line, it was considered as having sweet spot accuracy, while if it did not, then it was considered as not having sweet spot accuracy. For each spin level, the percentage of sweet spot accuracy was calculated for each player.

### 2.4. Data Processing

The TRAM-2 data were analyzed using MATLAB (R2020a). The original sampling rate of the two TRAM-2 rackets were not the same due to the microcontrollers having slightly different clock speeds at the high sampling rate, resulting in a different number of data points recorded over the fixed time duration. Therefore, for data analysis, all TRAM-2 data were first re-sampled at 8333 samples per second to ensure that all files had the same number of data points for data analysis. From the original file, timestamps (start and end times) associated with each correctly hit shot were first manually isolated. It was determined that the ball had been hit (compared to the air swings) when a threshold of 20 g was reached on the ICM20649 *z*-axis (perpendicular to the racket string bed) accelerometer sensor, between those time stamps. Time zero and impact were set when this threshold was met, with one second of data on either side of impact retained. The mean and standard deviation were calculated for all correctly hit shots for each spin level for each player. The players were then grouped, depending on both their playing ability level (experienced or recreational) and their preferred forehand grip position (Eastern or semi-Western). Data representing each group were then averaged, and the mean and standard deviation were calculated using the built-in MATLAB SPM1D “plot_meanSD” function. The ICM20649 and racket ADXL377 *x*-axis sensor data were adjusted to be negative for the left-handed players to match those of the right-handed players.

### 2.5. Analysing the Player’s Technique Using TRAM-2

The player’s technique was analyzed using TRAM-2 data to break down the stroke into the different phases (preparation, acceleration, impact, and follow-through), racket swing path angle (inverse tangent of racket ADXL377 *x*-axis over *z*-axis), racket face angle during the stroke (*y*-axis of the ICM20649 gyroscope), racket head acceleration (vector sum of the racket ADXL377 3-axes), and the player’s grip strength, forearm extensor, and flexor muscle activity as well as the vibrational data up the forearm (Table 1). The phases of the stroke were divided into early (first 50%) and late (last 50%).

To break down the stroke into the different phases, timestamps associated with selected movement were chosen using the ICM20649 sensor and the racket ADXL377 accelerometer. As previously described, the impact phase was determined as time zero. The start of the preparation phase and the end of the follow-through phase were determined when the racket head acceleration first reached above (Figure 2D(i)) and below (Figure 2D(ii)) the threshold of 1 g before and after impact, respectively. The inverse tangent of the racket ADXL377 *x*-axis over *z*-axis was used to determine the start of the acceleration phase when −90° was first reached, before impact (Figure 2E). This curve represents the angle of the acceleration vector relative to the direction the racket face is pointing over time, without the acceleration in the direction along the racket (*y*-axis). The −90° value represents the acceleration in the direction of the bottom edge of the racket and the pause in the swing before the racket moves forward towards impact. This vector direction data was also used to determine the racket low-to-high swing path during the acceleration phase of the stroke.

To process the EMG signal, a Butterworth Filter order 8 (high-pass (10 Hz) and low- pass (10 Hz) frequency cut-off) was applied to the ECR and FCR data. First, the 10 Hz high-pass filter was applied to the muscle data using the MATLAB command “filtfilt”, then the low-pass filter was applied to the absolute value of the high-pass filtered data using “filtfilt”. This provided the EMG signal amplitude over the isolated time period at 10 Hz.

Grip strength, ECR, and FCR activity were normalized to the maximum grip strength value and maximum voluntary contraction (MVC) activity level obtained across three maximum grip strength tasks. During these tasks, the player was required to maximally grip the racket using their preferred forehand grip position for 5 s in a comfortable seated position, with a 2 min rest between each test [69]. The middle two seconds were averaged for the grip strength, ECR, and FCR activity when the maximum grip strength value was recorded for each trial. All MVC tasks were performed between the guided warmup phase and the testing phase. The player testing procedure, as well as the MyoWare^®^ sensor and electrode placement, were the same as those in a study by Rigozzi, Cox [9].

The force transfer from impact up the forearm was calculated using the racket head acceleration and direction vector of the wrist ADXL377 3-axes and elbow ADXL377 3-axes accelerometers. A 400-Hz low-pass filter was applied to the acceleration signals to remove string vibration signals, and a 15-Hz high-pass was applied to eliminate the low-frequency components of the acceleration signal due to arm movements following impact [8,62]. For each player, the peak acceleration value recorded at the racket, wrist, and elbow after impact during the stroke were determined and expressed as a percentage of shock transfer from the racket to the wrist and the wrist to the elbow [62]. The percentage of shock transmission was calculated via [100 − (−((A_x_ − A_racket_)/A_racket_) × 100%)], where A_x_ represented the peak acceleration of the wrist or elbow, and A_racket_ represents the peak acceleration of the racket [62]. A larger percentage of shock transmission shows that more impact force was transferred from the racket to the arm, while a smaller percentage of shock transmission means that less impact force was transferred from the racket to the human arm [62].

### 2.6. Statistical Analysis

Samples were considered to be independent due to no significant difference (two-sample *t*-tests) between the demographic information and years of tennis experience between either the experienced and recreational or Eastern and semi-Western group comparisons. For the experienced vs. recreational and Eastern vs. semi-Western group comparisons, Lilliefors test for normality and subsequent paired (within groups with different spin levels) and two-sample (between groups with the same spin level) *t*-tests were used to compare the percentage of hits included (level of accuracy at completing the task) and the corresponding ball exit speed for the included hits. For the players included in the shock transfer analysis between the experienced and recreational players, the Lilliefors test for normality and subsequent paired (within groups with different spin levels) and two-sample (between groups with the same spin level) *t*-tests were used to compare the included hits, ball exit speed, ball spin rotation, sweet spot accuracy, grip force at impact, peak racket head acceleration, peak acceleration at the wrist, peak acceleration at the elbow, shock transfer from the racket to the wrist, and shock transfer from the racket to the elbow. Non-parametric Wilcoxon sign rank (paired *t*-test) and Wilcoxon rank sum (two-sample *t*-test) tests were used for comparisons where the assumption of normality was not met.

#### Statistical Parametric Mapping

Statistical parametric mapping (SPM) uses random field theory to make continuous comparisons across continuous time series data [70], and it is equivalent to the statistical methods used for discrete data series [71]. It allows the statistical results to be presented directly in the original sampling space, so their spatiotemporal biomechanical context is immediately apparent [72]. This method is being increasingly adopted by the biomechanics research community, especially in racket sports [14,64,71].

Two-sided SPM one-dimensional *t*-tests were used to compare the average data results of all players included within the experienced, recreational, Eastern, and semi-Western groups for both the flat and topspin spin levels. The comparisons were performed between the player’s racket face angle, racket head acceleration, grip strength, and ECR and FCR activity over the isolated 2 s stroke duration. For each variable, paired *t*-tests were used between the flat and topspin spin levels for the experienced, recreational, Eastern, and semi-Western groups, while two-sample *t*-tests were used for comparisons between the experienced and recreational players, as well as the Eastern and semi-Western players for the flat and topspin spin levels. Normality of the data was determined by simultaneously performing parametric and non-parametric SPM tests. If the results of both tests are identical, then parametric tests should be used; however, if the results vary, then non-parametric tests should be used [73]. The results showed that non-parametric tests should be used for all SPM comparisons performed. The positive or negative direction of the statistical non-parametric mapping (SnPM) result depends on the order of the variables included in the MATLAB SnPM *t*-test code [74]. For example, if the test compares Variable A (first input) to Variable B (second input), areas above zero imply that the mean of Variable A is higher than the mean of Variable B and vice versa for areas below zero [74]. If the SnPM {t} value crosses the threshold in either direction, and the area is shaded, it can be concluded that a significant difference has occurred (Figure 2F) [74]. Due to the high number of statistical analyses, the SnPM {t} results are visualized in a summarized manner, with the colored bars representing significant difference for that variable. All SPM analysis were performed in MATLAB (R2020a) using an open-source package located at https://spm1d.org (accessed on 4 April 2023). All statistical tests were two-sided and had a significance level of *p* < 0.05.

## 3. Results

TRAM-2 was able to successfully record the technique of all 40 players by simultaneously recording their racket face angle, racket head acceleration, grip strength, and forearm muscle activity under realistic playing conditions. Vibrational data was also recorded at the wrist and elbow for 37 players. Importantly, no players reported any issues with the device regarding their playing style.

### 3.1. Differences in Playing Experience—Experienced vs. Recreational

Compared to the recreational players, experienced players hit a significantly higher percentage of balls into the target area, while following the height guideline for both spin levels (Table 2). However, both groups hit a significantly lower percentage of balls into the target area for the flat spin level (54% experienced vs. 35% recreational) compared to the topspin level (81% experienced vs. 70% recreational). The average ball exit speed for the experienced players was significantly faster for the flat and topspin spin levels compared to that of the recreational players (Table 2). The flat ball exit speed was significantly faster than the topspin for both groups (Table 2).

**Figure 3:**
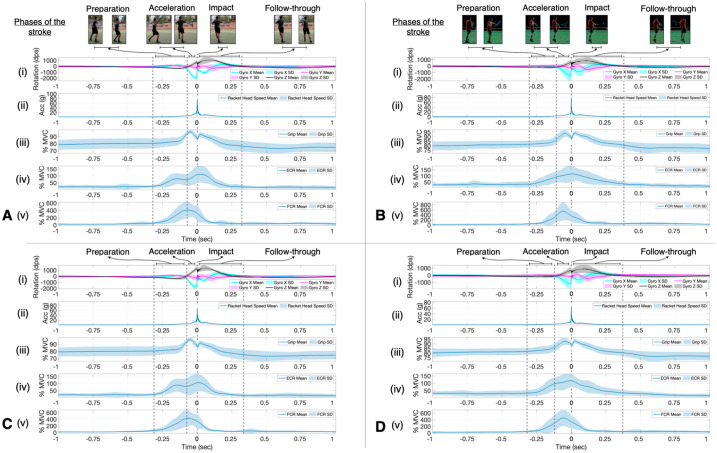
The mean (solid line) and standard deviation (colored area) of all the included hits shows changes in the (**A**) experienced flat (*n* = 18), (**B**) recreational flat (*n* = 22), (**C**) experienced topspin (*n* = 18), and (**D**) recreational topspin (*n* = 22) technique over the period of one second before and after impact (marked as time = 0 s). The player’s technique was analyzed using (i) gyroscope 3-axes, (ii) racket head acceleration (vector sum of the three racket ADXL377 accelerometer axes), (iii) grip strength, (iv) forearm extensor activity, and (v) forearm flexor activity. For the gyroscope, relative to the racket, the *x*-axis of the gyroscope (blue) was the direction across the string bed, the *y*-axis (purple) was the direction along the racket from the tip to the handle (also known as the racket face angle), and the *z*-axis (black) was perpendicular to the string bed. The duration of the different phases of the stroke are also shown.

Figure 3 shows the mean and standard deviation of the experienced (Figure 3A,C) and recreational (Figure 3B,D) player’s racket rotation (Figure 3A(i),B(i),C(i),D(i)), racket head acceleration (Figure 3A(ii),B(ii),C(ii),D(ii)), grip strength (Figure 3A(iii),B(iii),C(iii),D(iii)), forearm extensor (Figure 3A(iv),B(iv),C(iv),D(iv)), and flexor (Figure 3A(v),B(v),C(v),D(v)) muscle activity, and the duration of the different phases of the stroke can be seen in Figure 3 for both the flat (Figure 3A,B) and topspin (Figure 3C,D) spin levels. The impact of the ball on the racket strings can be seen in Figure 3A(ii),B(ii),C(ii),D(ii) where the peak racket head acceleration rapidly occurs, indicated at time equals zero. All players brushed the ball with the racket, moving in a low-to-high motion (Figure 3A(i),B(i),C(i),D(i) *z*-axis of the gyroscope) through the impact zone (−0.04–0.04 s), with a sudden change occurring at impact in all three axes of the gyroscope due to the vibrations from the ball hitting the strings (Figure 3A(i),B(i),C(i),D(i)). The grip strength pattern also changes throughout the whole stroke for all of the players, with the experienced players reaching their peak grip strength during the acceleration phase for both the flat (96.6% MVC) (Figure 3A(iii)) and topspin (96.4% MVC) (Figure 3C(iii)) spin levels. In contrast, the recreational players reached their peak grip strength value just after impact for both the flat (93.9% MVC) (Figure 3B(iii)) and topspin (93.0% MVC) (Figure 3D(iii)) spin levels.

Peak ECR activity occurred after impact for the experienced players and at impact for the recreational players, while peak FCR activity occurred during the acceleration phase for all players (Figure 3). The mean peak normalized muscle activity for the experienced players was 112.4% MVC (ECR) and 412.7% MVC (FCR) for the flat and 107.7% MVC (ECR), and 432.7% MVC (FCR) for the topspin spin level. For the recreational players, the mean peak normalized muscle activity was 117.1% MVC (ECR) and 543.9% MVC (FCR) for the flat and 117.6% MVC (ECR) and 455.0% MVC (FCR) for the topspin spin level.

**Figure 4:**
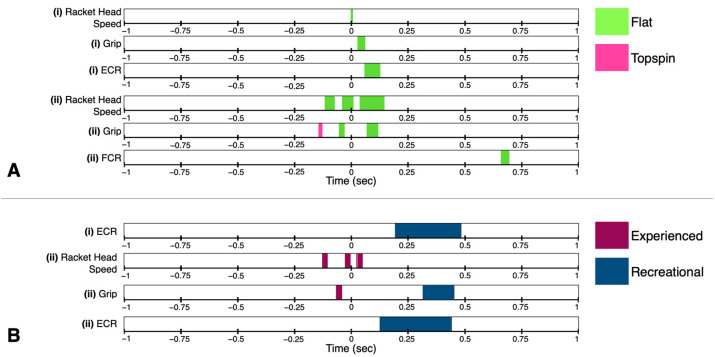
Simplified statistical non-parametric mapping analysis results are represented as colored areas over time between the experienced (*n* = 18, magenta) and recreational (*n* = 22, blue) playing level groups for both flat (green) and topspin (pink) spin levels. The color represents the variable which had a significantly higher mean in the comparison. The results are shown over the period of one second before and after impact (marked as time = 0 s). (**A**) Differences between flat and topspin spin levels between the (i) experienced group and the (ii) recreational group. (**B**) Differences between the experienced and recreational groups for the (i) flat spin level and the (ii) topspin spin level. Paired *t*-test were used between the flat and topspin spin level within each group, while two-sample *t*-tests were used for comparisons of the same spin level between groups (*p* < 0.05).

Because the duration of all strokes occurred less than 0.5 s before and after impact (Figure 3), only the statistically significant SnPM differences found within this window will be discussed. SnPM analysis revealed no significant difference in racket face angle between the flat and topspin spin level or in the flat or topspin spin levels between experienced and recreational players (Figure 4). The racket head acceleration was significantly higher at impact for both the experienced and recreational players in the flat spin level (Figure 4A). Recreational players also showed significantly higher racket head acceleration during part of the acceleration and early follow-through phases compared to that occurring during the topspin spin level (Figure 4A(ii)). The experienced players showed a significantly higher racket head acceleration during the topspin spin level compared to the recreational players during part of the late preparation and acceleration phases, as well as during the early follow-through phase (Figure 4B(ii)). Both experienced and recreational players showed significantly higher grip strength during part of the early follow-through phase of the flat spin level compared to that during the topspin (Figure 4A). During the acceleration phase, the recreational players first gripped the racket significantly higher during the topspin spin level, as well as during the flat spin level closer to impact (Figure 4A(ii)). However, during the topspin spin level, the experienced players gripped the racket significantly tighter during part of the acceleration phase, while the recreational players gripped the racket significantly higher during part of the late follow-through phase (Figure 4B(ii)). The experienced players had a significantly higher level of ECR activity during part of the early follow-through phase of the flat spin level (Figure 4A(i)). However, the recreational players had a significantly higher level of ECR activity during part of the follow-through phase for both the flat and topspin spin levels compared to the experienced players (Figure 4B). There were no significant differences observed in the FCR activity for either group of players at either spin level.

**Figure 5:**
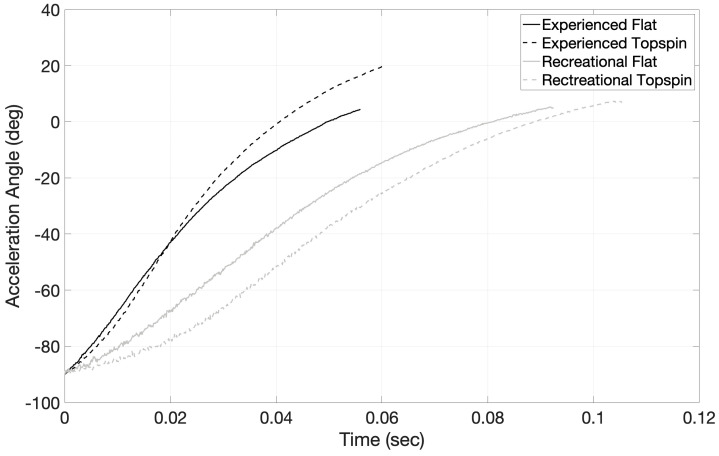
The racket low-to-high swing path acceleration angle (inverse tangent of racket ADXL377 *x*-axis over *z*-axis) is shown for the acceleration phase of the stroke for the experienced flat (*n* = 18, black [^___^]), experienced topspin (*n* = 18, black [- - -]),), recreational flat (*n* = 22, grey [^___^]), and recreational topspin (*n* = 22, grey [- - -]) groups. The duration of the acceleration phase is shown on the *x*-axis (time), while the change in rotation angle (*y*-axis) represents the amount of low-to-high swing path (brushing action) occurring for each group of players. The figure shows the experienced players exhibiting a shorter acceleration phase than the recreational players, with the experienced topspin group having the largest low-to-high brushing motion.

The duration of the acceleration phase can be seen in Figure 5 for the flat and topspin spin levels for both the experienced and recreational players. The experienced players hit the ball with significantly more low-to-high swing movement during the topspin (−90° to 20.0°) compared with during the flat (−90° to 4.3°) spin levels (Figure 5). In comparison, the recreational players hit using a similar level of low-to-high swing movement during both the flat (−90° to 5.2°) and topspin (−90° to 6.8°) spin levels (Figure 5). The differences in the duration of the acceleration phase can also be seen in Figure 5, with the experienced flat group having the shortest duration, followed by the experienced topspin group, the recreational flat group, and the recreational topspin group.

### 3.2. Shock Transfer Frame Vibrations from Racket to Wrist and Racket to Elbow—Experienced vs. Recreational

Players in the experienced group (*n* = 17) hit the ball significantly faster than the recreational players (*n* = 20) for both spin levels, with all players hitting the flat spin level significantly faster than the topspin spin level (Table 3). The topspin spin level also had a significantly higher level of ball spin rotation compared to the flat spin level for both groups, with the experienced topspin spin level having a significantly higher level of ball spin rotation compared to the recreational players topspin spin level (Table 3). The experienced players also had a significantly higher level of sweet spot accuracy for both spin levels compared to the recreational players, with the topspin spin level showing a significantly higher level of accuracy compared to the flat spin level for all players (Table 3). Interestingly, there was no significant difference between any of the comparisons for the grip strength force at impact. However, the peak racket head acceleration (frame vibrations only) was significantly higher for the flat spin level for both groups compared to the topspin spin level, while the peak acceleration at the wrist and elbow were significantly higher for the experienced players compared to the recreational players for both spin levels (Table 3). Additionally, there was a significantly higher percentage of shock transfer from the racket to the wrist for the topspin spin level compared to the flat spin level for both groups, with the experienced players having a higher percentage for both spin levels compared to the recreational players (Table 3). However, there was no statistically significant difference in shock transfer from the racket to the elbow between either spin level within the experienced and recreational players, although the experienced player topspin spin level showed a significantly higher level of shock transfer than the recreational player topspin level (Table 3).

### 3.3. Differences in Preferred Forehand Grip Position—Eastern vs. Semi-Western

Within the Eastern and semi-Western grip position groups, there was a significantly higher level of hits included in the topspin spin level compared to the flat for both groups (Table 4). Additionally, there was a significantly higher ball exit speed for the flat spin level compared to the topspin spin level for both the Eastern and semi-Western groups (Table 4). Additionally, there was no significant difference between the two grip position groups for any of the comparisons seen in Table 4.

**Figure 6:**
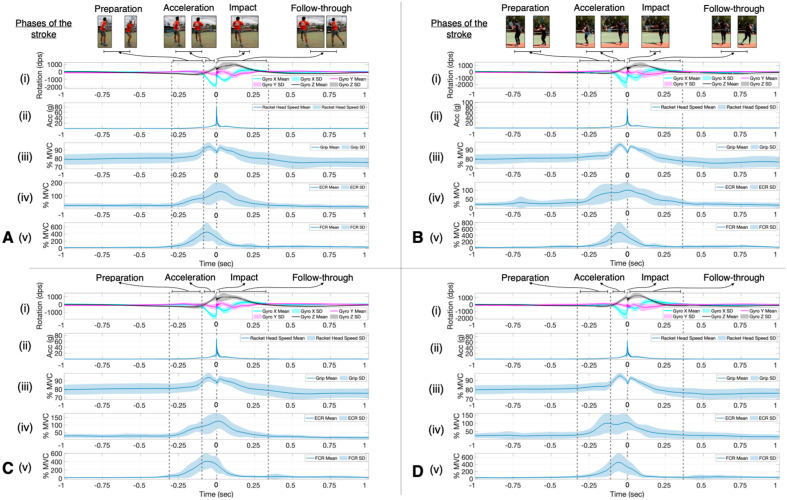
The mean (solid line) and standard deviation (colored area) of all the included hits show changes in the (**A**) Eastern flat (*n* = 21), (**B**) semi-Western flat (*n* = 17), (**C**) Eastern topspin (*n* = 21), and (**D**) semi-Western topspin (*n* = 17) groups’ technique over the period of one second before and after impact (marked as time = 0 s). The player’s technique was analyzed using (i) gyroscope 3-axes, (ii) racket head acceleration (vector sum of the three racket ADXL377 accelerometer axes), (iii) grip strength, (iv) forearm extensor activity, and (v) forearm flexor activity. For the gyroscope, relative to the racket, the *x*-axis of the gyroscope (blue) was the direction across the string bed, the *y*-axis (purple) was the direction along the racket from the tip to the handle (also known as the racket face angle), and the *z*-axis (black) was perpendicular to the string bed. The duration of the different phases of the stroke are also shown.

The mean and standard deviation of the Eastern (Figure 6A,C) and semi-Western (Figure 6B,D) players’ racket rotation (Figure 6A(i),B(i),C(i),D(i)), racket head acceleration (Figure 6A(ii),B(ii),C(ii),D(ii)), grip strength (Figure 6A(iii),B(iii),C(iii),D(iii)), forearm extensor (Figure 6A(iv),B(iv),C(iv),D(iv)), and flexor (Figure 6A(v),B(v),C(v),D(v)) muscle activity and duration of the different phases of the stroke can be seen in Figure 6 for both the flat and topspin spin levels. Similar trends as were observed in Figure 3 can be seen in Figure 6 for all variables, except that the peak grip strength was reached during the acceleration phase for all players, and the peak ECR activity was reached just after and just before impact for the flat and topspin levels, respectively, for the semi-Western grip players, while the peak ECR activity occurred just after impact for the Eastern grip players.

**Figure 7:**
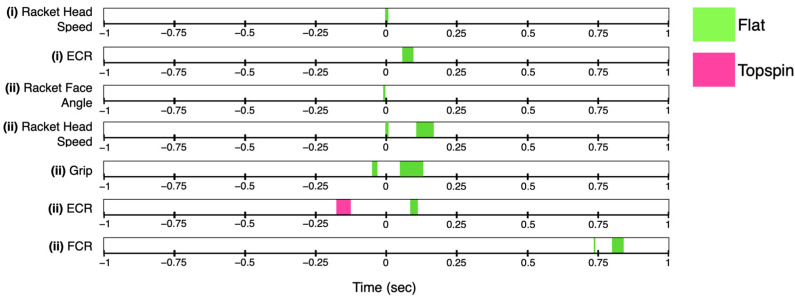
The simplified statistical non-parametric mapping analysis results are represented as colored areas over time between the Eastern (*n* = 21) and semi-Western (*n* = 17) preferred forehand grip position groups for both flat (green) and topspin (pink) spin levels. The color represents the variable which exhibited a significantly higher mean in the comparison. The results are shown over the period of one second before and after impact (marked as time = 0 s). The differences between the flat and topspin spin levels between the (i) Eastern and the (ii) semi-Western groups are shown. Paired *t*-test were used between the flat and topspin spin level within each group, while two-sample *t*-tests were used for comparisons of the same spin level between groups (*p* < 0.05). No significant differences were found between the Eastern and semi-Western groups for any of the comparisons.

For the SnPM analysis, the Eastern group had a significantly higher racket head acceleration around impact for the flat spin level compared to the topspin spin level (Figure 7(i)). Additionally, the Eastern group showed a significantly higher ECR activity during part of the early follow-through phase of the flat spin level compared to the topspin level (Figure 7(i)). For the semi-Western group, the flat spin level showed a significantly higher level of racket face rotation just prior to impact, as well as higher grip strength during part of the acceleration and early follow-through phases compared to those for the topspin spin level (Figure 7(ii)). The ECR activity changed for the semi-Western group, with the topspin spin level having a significantly higher level of activity just before the acceleration phase, and the flat spin level having a significantly higher level of activity during part of the early follow-through phase (Figure 7(ii)). There were no significant differences observed in the FCR activity for either group of players for both spin levels.

## 4. Discussion

TRAM-2 was able to demonstrate that all players have a unique forehand stroke technique under realistic playing conditions. Kinematic motion analysis associated with each player’s technique was successfully recorded for 40 players using TRAM-2. As compared to the recent work of Rigozzi, Vio [16], this current study included double the number of participants, and TRAM-2 recorded nearly three times the amount of kinematic data than did previous studies using wearable technology for player motion analysis in racket sports under realistic playing conditions. Using TRAM-2, we found that the level of playing experience, the preferred forehand grip position, and the level of spin with which the player hit the ball all have a significant influence on the player’s racket face angle, racket head acceleration, grip strength, ECR activity, and shock transfer from the racket to the wrist and elbow during the forehand stroke. This supports our hypothesis that regardless of the level of playing experience, TRAM-2 can successfully be used to compare and identify unique injury development risk factors for the forehand stroke associated with tennis players’ technique under realistic playing conditions.

### 4.1. Player Technique Analysis

Our results showed that players found it easier to complete the topspin spin level compared to flat spin level. This is unsurprising, given the natural tendency for players to prefer to hit the ball with topspin instead of flat to increase the margin of error of getting the ball over the net. Even though there was a significantly higher level of ball spin rotation for all players for the topspin spin level compared to the flat, there was no significant difference in racket face angle between either spin level. These results contrast with those of Kwon, Pfister [13], who found that as the racket face angle became more closed, the level of ball spin increased. However, their study required their participants to hit forehand shots at shoulder height in a laboratory environment, straight towards a target area, compared to our method, in which the players hit the ball cross-court freely, under realistic playing conditions.

Significantly higher racket head acceleration at impact and faster ball exit speed were found for the flat spin level compared to the topspin spin level for all players. Our ball exit speed results support those of previous studies showing that the flat spin level exhibits a faster ball exit speed than the topspin spin level [10,37,40], potentially due to faster racket head speed resulting in greater ball exit speed during the forehand stroke [31]. In contrast, Rogowski, Rouffet [36] found no significant difference in the resultant racket face velocity at impact between the flat and topspin spin levels. This difference could potentially be due to the ball exit speed not being measured and their study involving junior players (under the age of 15) who might not have been able to develop the same level of racket head acceleration as the adults in the current study.

The duration of the swing was within one second for all players, with the experienced players completing the acceleration phase of the stroke approximately twice as fast as the recreational players. All players brushed the ball with the racket, moving in a low-to-high brushing motion (wrist abduction) during the acceleration phase, with the experienced players having the highest level during their topspin spin level, followed by the recreational topspin, recreational flat, and experienced flat spin levels. Previous studies have also found a higher level of wrist abduction during the topspin forehand drive compared to the flat [38,40], with approximately 6% of the racket head speed contributed by hand abduction during a cross-court forehand stroke [64]. This is important because wrist abduction uses both the ECR and FCR muscles to generate topspin on the ball [39]. Surprisingly, our results show no significant difference in ECR or FCR activity during the acceleration and impact phases for all players during both spin levels. This shows that even though there is a difference in the low-to-high swing movement between players and spin levels, this difference may not be large enough to result in a significant difference in forearm muscle activity. Nevertheless, our results show that TRAM-2 can be used as a training tool to identify how much low-to-high swing path the player is using during their technique, as well as, potentially, the amount of topspin generated on the ball through this motion.

### 4.2. Forehand Grip Position

Kinematic parameters associated with a players technique are often not analyzed with respect to the different forehand grip positions of the players [40], and the forehand grip position of the participants is not typically reported. This is the first study to statistically analyze the player’s preferred forehand grip position based on the specific hand markings on the racket handle at the end of the testing session. Of our 40 players, 21 players preferred Eastern, 17 players preferred semi-Western, and 2 players preferred Continental forehand grip positions. Interestingly, our study was conducted in Australia, while in other forehand studies conducted in Europe the choice of Eastern forehand grip position was less common. For example, only 2 of the 21 players who participated in the study by Rota, Hautier [41], and only 1 of the 13 players in the study by Landlinger, Lindinger [75], used an Eastern forehand grip position. All other participants used either a semi-Western or Western forehand grip position. Thus, it appears that the preference of forehand grip position taught by coaches in the modern game may vary with geography, or methods used to determine the player grip position may not have been accurate.

Repetitive loading during tennis strokes has led to an increase in players suffering from wrist injuries in the modern game [45]. The forehand grip position influences the alignment of the wrist at impact and the kinematics of the forehand stroke and generation of topspin, which then influences the behavior of the ball after impact [42]. At ball impact, the wrist is hyperextended [35] and is in maximal ulnar deviation, putting the extensor carpi ulnaris (ECU) tendon at increased risk of injury [14]. We found no significant difference in racket face angle at impact between the Eastern and semi-Western grip positions. This is of note, given that these grips differ in the alignment of the hand on the handle of the racket. Coaches consider that the changes in natural racket face orientation at impact may allow for easier generation of topspin on the ball [43]. Tagliafico, Ameri [6] found that ulnar-sided wrist injuries (including the ECU tendon) were associated with the semi-Western or Western grip, and radial-sided injuries (including the FCR tendon) were associated with the Eastern grip. However, we found no significant difference in ECR or FCR activity between either the Eastern or semi-Western grip positions for both spin levels. Interesting, all injured players in the study by Tagliafico, Ameri [6] self-reported using a synthetic gut string, while players in our study used a polyester string. This suggests that the string type used by the player, rather than the preferred forehand grip position or spin level played on the ball, may have a role in the risk of developing a wrist injury.

### 4.3. Elbow Tendinopathy

#### 4.3.1. Forearm Extensor and Flexor Muscle Activity

LET is more common in recreational players [49], with the repetitive overload of the forearm extensor muscles suggested as a cause of LET development in tennis players [4,51]. Our results showed that recreational players demonstrated significantly higher ECR activity than experienced players during most of the follow-through phase at both spin levels. A study by Morris, Jobe [29] found that the ECR muscles exhibited high activity in all strokes. Therefore, the higher level of ECR activity observed in the current study potentially puts recreational players at increased risk of LET compared to experienced players due to the repetitive nature of the forehand stroke.

Our results showed that peak ECR activity occurred after impact for the experienced and at impact for the recreational players, while peak FCR activity occurred during the acceleration phase for both the experienced and recreational players. We also found that the effect of spin level on ECR activity meant that experienced players had a significant increase in ECR activity during part of the early follow-through phase during the flat spin level compared to the topspin level. These results contrast with those found in studies by Rogowski, Rouffet [36] who analyzed the average ECR and FCR% MVC activity during the acceleration phase of the forehand stroke, as well as with the results of our previous study [10], in which we analyzed peak ECR and FCR% MVC activity associated with each forehand hit. These studies found that ECR muscle activity significantly decreased from the flat to topspin spin level in higher level players during the forehand stroke. The discrete statistical analysis performed by Rogowski, Rouffet [36] and in our previous study [10], compared to the continuous statistical analysis performed in this study may explain the differences observed due to peak ECR values potentially occuring at different stages of the stroke for each player. However, our results also showed no significant difference in ECR activity for recreational players between either spin level, which support the findings of Rogowski, Rouffet [36] for less experienced players.

We observed no significant difference in FCR activity for all players between either the flat or topspin spin levels, level of playing ability, or preferred forehand grip position throughout the forehand stroke. These findings contrast with those of Rogowski, Rouffet [36] who found a significant decrease in FCR activity from the flat to the topspin spin level in junior players, who were able to increase the vertical velocity of the racket face at impact by more than 150% when hitting from flat to topspin. However, our results support our previous findings of no significant difference in peak FCR activity between flat and topspin spin levels in experienced players [10]. This is somewhat surprising, given that the forehand stroke is typically associated with the development of MET [49], due to the key role of FCR activation in wrist flexion [39], the intentional motion players have been reported to use to increase ball exit speed [2] and produce topspin on the forehand stroke [38]. However, the study by Rogowski, Rouffet [36] was performed using junior players who would have had less developed muscle forearm groups compared to the adult players in our previous and current studies. With less developed muscle groups, significant EMG signals produced by neighboring muscles can influence the recorded electrode readings [76].

#### 4.3.2. Shock Transfer—Frame Vibrations

Our results showed that all players had a significantly higher level of sweet spot accuracy and a significantly lower level of peak racket head acceleration (frame vibrations) for the topspin spin level compared to the flat spin level. Although the accuracy of the Head Tennis Sensor^®^ cannot be confirmed [27], our results support the racket vibrating less when the ball is hit along the curved vibration node on the racket (sweet spot accuracy) [63], especially under realistic playing conditions. The ball exit speed for all players was significantly higher on the flat spin level compared to the topspin, with the experienced players having a significantly higher level of ball exit speed compared to the recreational players. Faster ball exit speed increases the number of vibrations at the racket and wrist, but has a minimal effect on their frequency [77]. We observed significantly higher peak frame vibrations at the racket for the flat level compared to the topspin spin level for all players, in line with faster ball exit speed leading to higher frame vibrations. However, significant differences in peak frame vibrations at the racket were only seen between the two spin levels within the groups, rather than between the two groups for the same spin.

Our findings support previous research findings that shock impact is highest at the racket, followed by the wrist, and then the elbow during tennis strokes [8,62]. There was a significantly higher level of shock transfer from the racket to the wrist for the experienced players flat and topspin spin levels than for the recreational players, with the topspin spin level having a significantly higher amount of shock transfer than the flat spin level. There was also a significantly higher level of ball spin rotation for the topspin spin level compared to the flat level for all players and for the experienced topspin level compared to the recreational topspin level. These results show that hitting the ball with topspin potentially leads to more shock transfer from the racket to the wrist, especially for higher level players.

In terms of shock transfer from the racket to elbow, approximately 3.5% was transferred for experienced players and 2.5% for the recreational players. The only significant level of shock transfer occurred with experienced players at the topspin level. Interesting, even though the ECR and FCR activity was above 100% MVC at impact, there was no significant difference in ECR and FCR activity between the experienced and recreational players for either spin level during the impact and the early follow-through phases. These findings contradict the concept that when the skeletal muscles stiffen near maximum contraction, the vibrations from impact are transferred efficiently and directly to the myotendinous junction, causing repeated microtrauma [8,54].

#### 4.3.3. Grip Strength

The dynamic behavior of the racket is highly dependent on the player’s grip force [78]. Our results showed that players have unique grip force patterns throughout the stroke. The experienced players gripped the racket the tightest (over 96% MVC) during the acceleration phase, and the recreational players gripped the racket the tightest (over 93% MVC) just after impact. All players showed a similar level of grip strength at impact. A study by Knudson and White [56] also revealed players to have similar rhythmic gripping patterns using data recorded at the base of the index finger. However, their study recorded forces created by the hand at two sensor locations on the handle, while our pressure sensor measured only one output from the whole hand. Another study by Schnabel and Hennig [67] compared the grip strength of expert and casual players during the forehand stroke and found that the expert players released their peak grip strength prior to impact significantly earlier than did the casual players, and they experienced a significantly lower peak acceleration load at the wrist. However, our results contrast with these findings, showing no significant difference in grip strength found around impact between the experienced and recreational players, and the experienced players had significantly higher levels of peak acceleration at the wrist compared to the recreational players. Differences in the results between the two studies could be due to the ball exit speed and spin level not being recorded in the study by Schnabel and Hennig [67]. However, our results clearly suggest that grip strength at impact does not influence the amount of frame vibrations generated at the racket or transferred to either the wrist or elbow during the forehand stroke under realistic playing conditions.

### 4.4. Limitations and Future Directions

Our results showed that for both spin levels, the peak FCR% MVC values were higher than the peak ECR% MVC values for all players. This results contrast to those of previous studies where ECR% MVC activity was found to be higher than FCR% MVC activity [10,36,79]. These differences may be due to the different protocols for the normalization task. Normalization of the EMG amplitude provides information about the magnitude of muscle activation relative to a reference value [69]. Both the maximum voluntary isometric contraction task specific to the isolated muscle [10,36,79] and the maximum EMG activity level obtained during the maximum grip strength task used in the current study are acceptable methods of EMG normalization [69]. This method was chosen for the current study so that changes in ECR and FCR activity could be observed over time relative to the player’s grip strength. However, it should be noted that the players were not required to keep their wrists in a fixed position during the normalization task, potentially resulting in lower FCR normalization values due to players having relied more on their ECR muscles to provide the maximum grip strength [80].

SPM analysis requires temporal data normalization [71]. Therefore, another study limitation was the threshold of 20 g used to represent impact and align the data. This threshold did not take into consideration individual players having different levels of racket head acceleration during the acceleration phase of the stoke (Figure 5), especially with the high sampling rate of TRAM-2. In future studies, researchers should consider differences in racket head acceleration between players when performing temporal data normalization for SPM analysis in racket sports. Additionally, we were not able to perform SPM analysis within the experienced and recreational groups for the different preferred forehand grip positions due to player numbers not reaching the recommended minimum of 8 players per group [81]. Therefore, future studies should consider larger player numbers to meet this minimum recommended requirement.

It has been suggested that frequencies in the range of 80–200 Hz are likely to contribute to the development of elbow tendinopathy in tennis players [8]. Vibrations generated by the racket during the shot also greatly contribute to how the racket feels to each player [82], with the first bending mode of the racket transmitted into the players forearm [66]. The player’s grip force intensity also influences the first two bending modes and the first torsional mode frequencies of the racket [66]. Therefore, potential future directions include performing a spectral analysis on the acceleration data of the experienced and recreational players; if the racket feels different to players, it could provide insight into whether one group is at increased risk of elbow tendinopathy development.

Improper stroke technique can result in potential injury due to joints becoming overloaded when the direction of energy flow is not efficiently transferred from one joint to another along the kinetic chain [5]. Another future direction is to use TRAM-2 to analyze other strokes (except double-handed backhand, due to the positioning of the microcontroller on the racket handle) under realistic playing conditions. For example, the single-handed backhand is commonly associated with the development of LET in recreational players due to eccentric loading [49]. Therefore, TRAM-2 could be used to measure the player’s technique, and it could also be used as an individualized training tool to potentially reduce the players’ risk of developing elbow tendinopathy by reducing their grip strength, forearm muscle activity, and shock transfer.

## 5. Conclusions

Players were required to hit 40 forehand strokes cross-court, following the height guideline, into the target zone for each forehand spin level (flat and topspin), and their preferred forehand grip position was noted. This study demonstrates that it is possible to make, design, and validate a wearable device which can simultaneously measure the risk factors associated with elbow injury development under realistic playing conditions in players of all levels of experience. TRAM-2 was able to demonstrate that all players have a unique forehand stroke technique, and that the level of playing experience, preferred forehand grip position, and level of spin with which the player hits the ball all have a significant influence on the players racket face angle, racket head acceleration, grip strength, ECR activity, and shock transfer from the racket to wrist and elbow during the forehand stroke.

All players had a similar level of grip strength at impact, and grip strength at impact does not influence the amount of frame vibrations generated at the racket or transferred to either the wrist or elbow during the forehand stroke under realistic playing conditions. Interestingly, the preferred forehand grip position did not significantly influence any of the comparisons. However, the experienced players’ topspin spin level had the highest ball spin rotation, low-to-high action, and shock transfer to the wrist and elbow. This suggests that hitting the ball with higher levels topspin potentially increases the amount of shock transferred from the racket along the forearm to the elbow. Additionally, recreational players exhibited significantly higher ECR activity during most of the follow-through phase compared to experienced players for both spin levels. Given that elbow tendinopathy development has been associated with repetitive overuse and shock transfer, experienced players hitting the ball with higher levels of topspin and recreational players relying on their ECR muscles at the completion of the stroke could potentially contribute to an increased risk of developing elbow tendinopathy.

## Figures and Tables

**Table 1:** The parameters derived from the Head Tennis Sensor^®^ (ball exit speed (kph), ball spin rotation (rotation per minute (rpm)) and impact location of the ball on the racket face corresponding to each hit) and TRAM-2—breaking down the stroke into the different phases (preparation, acceleration, impact, and follow-through), racket swing path angle, racket face angle during the stroke, racket head acceleration and the player’s grip strength, forearm extensor and flexor muscle activity, as well as the vibrational data up the forearm—devices, as well as how these parameters were derived.

	Parameter	How It Was Derived	
**Head Tennis Sensor^®^**	Ball exit speed (km/h)	Accompanying mobile phone application	
	Ball spin rotation (rpm)	Accompanying mobile phone application	
	Ball impact location on the racket face	Accompanying mobile phone application	
**TRAM-2**	Different phases of the stroke (preparation, acceleration, impact and follow-through)	Preparation	The start of the phase was determined when the racket head acceleration first reached above the threshold of 1 g before impact
		Acceleration	The start of the phase was determined when the inverse tangent of racket ADXL377 *x*-axis over *z*-axis first reached −90° before impact
		Impact	Was determined when a threshold of 20 g was reached on the ICM20649 *z*-axis
		Follow-through	The end of the phase was determined when the racket head acceleration first reached below the threshold of 1g after impact
	Racket swing path angle	Inverse tangent of racket ADXL377 *x*-axis over *z*-axis	
	Racket face angle	*Y*-axis of the ICM20649 gyroscope	
	Racket head acceleration	Vector sum of the racket ADXL377 3-axes	
	Grip strength	Recorded using the custom built pressure sensor surrounding racket handle and normalized to the maximum grip strength value	
	Forearm extensor muscle activity	Recorded using the ECR MyoWare^®^ device and normalized to the maximum voluntary contraction	
	Forearm flexor muscle activity	Recorded using the FCR MyoWare^®^ device and normalized to the maximum voluntary contraction	
	Vibrational data up the forearm	Calculated using the racket head acceleration and direction vector of the wrist ADXL377 3-axes and elbow ADXL377 3-axes	

**Table 2:** The demographic information (age, height, weight, and years of playing experience) for the experienced (*n* = 18) and recreational (*n* = 22) playing level groups. Additionally, the number of hits included (accuracy of the player hitting the ball into the target area following the spin height guideline) for each spin level, as well as the ball exit speed corresponding to the included hits of the experienced and recreational players, are shown. Statistical significance is marked for comparisons between the flat and topspin spin level within the group (* paired *t*-test (*p* < 0.05)) and the same spin level between the two groups (^ two-sample *t*-test (*p* < 0.05)).

	Experienced (*n* = 18)		Recreational (*n* = 22)	
	Flat	Topspin	Flat	Topspin
**Age (Mean ± SD)**	32.9 ± 10.6		36.4 ± 12.8	
**Height (Mean ± SD)**	180.6 ± 5.7		178.5 ± 8.1	
**Weight (Mean ± SD)**	81.6 ± 9.4		78.2 ± 10.5	
**Years of Tennis Experience (Mean ± SD)**	24.7 ± 10.9		19.1 ± 11.6	
**Hits included in spin level (%) (Mean ± SD)**	54.0 ± 13.0 * ^	80.6 ± 8.1 * ^	35.1 ± 15.0 * ^	69.5 ± 15.1 * ^
**Ball Exit Speed (km/h) (Mean ± SD)**	124.4 ± 13.0 * ^	109.9 ± 13.7 * ^	110.6 ± 9.4 * ^	94.1 ± 10.1 * ^

**Table 3:** The ball exit speed, ball spin rotation, sweet spot accuracy, grip force at impact, peak racket head acceleration (vector sum frame vibrations), peak acceleration at the wrist (vector sum frame vibrations), peak acceleration at the elbow (vector sum frame vibrations), and percentage of shock transfer from the racket to the wrist and the racket to the elbow for the flat and topspin spin level of the experienced (*n* = 17) and recreational (*n* = 20) groups. Statistical significance is marked for comparisons between the flat and topspin spin level within the group (* paired *t*-test (*p <* 0.05)), the same spin level between the two groups (^ two-sample *t*-test (*p* < 0.05)), and non-parametric tests (* ^), respectively.

	Experienced (*n* = 17)		Recreational (*n* = 20)	
	Flat	Topspin	Flat	Topspin
**Ball Exit Speed (km/h) (Mean ± SD)**	124.9 ± 12.3 * ^	110.8 ± 13.2 * ^	111.1 ± 9.1 * ^	94.3 ± 10.0 * ^
**Ball Spin Rotation (rpm) (Mean ± SD)**	1332.5 ± 300.8 *	2129.9 ± 432.4 * ^	1361.3 ± 291.8 *	1592.2 ± 373.8 * ^
**Sweet Spot Accuracy (%) (mean ± SD)**	52.3 ± 22.4 * ^	70.9 ± 15.6 * ^	30.6 ± 19.4 * ^	49.1 ± 30.0 * ^
**Grip Strength at Impact (%MVC) (Mean ± SD)**	89.4 ± 4.0	89.3 ± 4.3	90.2 ± 2.8	90.3 ± 2.4
**Peak Racket Head Speed (g) (Mean ± SD)**	61.4 ± 7.3 *	49.8 ± 7.5 *	61.6 ± 6.4 *	49.6 ± 5.3 *
**Peak Acceleration at Wrist (g) (Mean ± SD)**	9.8 ± 2.5 ^	11.0 ± 4.5 ^	7.7 ± 2.3 ^	7.7 ± 2.4 ^
**Peak Acceleration at Elbow (g) (Mean ± SD)**	1.9 ± 0.9 ^	1.7 ± 0.7 ^	1.2 ± 0.5 ^	1.2 ± 0.5 ^
**Shock Transfer Racket to Wrist (%) (Mean ± SD)**	16.3 ± 5.0 * ^	22.0 ± 7.5 * ^	12.6 ± 3.9 * ^	15.8 ± 5.5 * ^
**Shock Transfer Racket to Elbow (%) (Mean ± SD)**	3.2 ± 1.7	3.4 ± 1.3 ^	2.1 ± 1.2	2.4 ± 1.1 ^

**Table 4:** The demographic information (age, height, weight, and years of playing experience) for the Eastern (*n* = 21) and semi-Western (*n* = 17) preferred forehand grip position groups. Additionally, the number of hits included (accuracy of the player hitting the ball into the target area following the spin height guideline) for each spin level and ball exit speed corresponding to the included hits of the Eastern and semi-Western grips are shown. Statistical significance is marked for comparisons between the flat and topspin spin level within the group (* paired *t*-test (*p* < 0.05)). There was no statistical significance for comparisons between the same spin level between the two groups (two-sample *t*-test (*p* < 0.05)).

	Eastern (*n* = 21)		Semi-Western (*n* = 17)	
	Flat	Topspin	Flat	Topspin
**Age (Mean ± SD)**	38.1 (13.5)		30.9 (9.2)	
**Height (Mean ± SD)**	179.2 (6.5)		180.0 (8.3)	
**Weight (Mean ± SD)**	80.5 (9.2)		79.0 (11.7)	
**Years of Tennis Experience (Mean ± SD)**	24.6 (13.3)		18.6 (8.5)	
**Hits included in spin level (%) (Mean ± SD)**	47.5 (15.5) *	76.3 (11.8) *	40.6 (17.4) *	73.2 (15.8) *
**Ball Exit Speed (km/h) (Mean ± SD)**	116.6 (12.5) *	101.9 (14.3) *	119.5 (11.7) *	102.3 (14.0) *

## Data Availability

Data supporting the reported results are available upon request to the authors.

## References

[1] Reid M., Morgan S., Whiteside D. (2016). Matchplay characteristics of Grand Slam tennis: Implications for training and conditioning. J. Sport. Sci..

[2] Seeley M.K., Funk M.D., Denning W.M., Hager R.L., Hopkins J.T. (2011). Tennis forehand kinematics change as post-impact ball speed is altered. Sport. Biomech..

[3] Shannon N., Cable B., Wood T., Kelly IV J. (2020). Common and less well-known upper-limb injuries in elite tennis players. Curr. Sport. Med. Rep..

[4] Field L.D., Altchek D.W. (1995). Elbow injuries. Clin. Sport. Med..

[5] Chung K.C., Lark M.E. (2017). Upper extremity injuries in tennis players: Diagnosis, treatment, and management. Hand Clin..

[6] Tagliafico A.S., Ameri P., Michaud J., Derchi L.E., Sormani M.P., Martinoli C. (2009). Wrist injuries in nonprofessional tennis players: Relationships with different grips. Am. J. Sport. Med..

[7] Ciccotti M.C., Schwartz M.A., Ciccotti M.G. (2004). Diagnosis and treatment of medial epicondylitis of the elbow. Clin. Sport. Med..

[8] Hennig E.M., Rosenbaum D., Milani T.L. (1992). Transfer of tennis racket vibrations onto the human forearm. Med. Sci. Sport. Exerc..

[9] Rigozzi C.J., Cox J., Vio G.A., Poronnik P. (2022). Simultaneous measurement of forearm muscle activity, vibrational transfer and grip strength during the tennis forehand stroke using a novel wearable device-a pilot study. Proceedings of the 2022 IEEE International Workshop on Sport, Technology and Research (STAR).

[10] Rigozzi C., Cox J., Vio G.A., Martens W.L., Poronnik P. (2022). The effect of spin level and ball exit speed on forearm muscle activity in the tennis forehand stroke. Int. J. Sport. Sci. Coach..

[11] Ahmadi A., Rowlands D.D., James D.A. (2010). Development of inertial and novel marker-based techniques and analysis for upper arm rotational velocity measurements in tennis. Sport. Eng..

[12] Iijima Y., Watanabe K., Kobayashi K., Kurihara Y. Measurement and analysis of tennis swing motion using 3D gyro sensor. Proceedings of the SICE Annual Conference.

[13] Kwon S., Pfister R., Hager R.L., Hunter I., Seeley M.K. (2017). Influence of tennis racquet kinematics on ball topspin angular velocity and accuracy during the forehand groundstroke. J. Sport. Sci. Med..

[14] Loushin S.R., Kakar S., Tetzloff S.U., Lubbers P., Ellenbecker T.S., Kaufman K.R. (2022). Upper extremity kinematics and electromyographic activity in uninjured tennis players. Appl. Sci..

[15] Ikenaga M., Okuma N., Nishiyama H., Chiba S., Nishino K., Omori G., Nunome H. (2020). Influence of Ball Impact Location on Racquet Kinematics, Forearm Muscle Activation and Shot Accuracy During the Forehand Groundstrokes in Tennis. Proceedings.

[16] Rigozzi C.J., Vio G.A., Poronnik P. (2022). Application of wearable technologies for player motion analysis in racket sports: A systematic review. Int. J. Sport. Sci. Coach..

[17] Cust E.E., Sweeting A.J., Ball K., Robertson S. (2019). Machine and deep learning for sport-specific movement recognition: A systematic review of model development and performance. J. Sport. Sci..

[18] Aroganam G., Manivannan N., Harrison D. (2019). Review on wearable technology sensors used in consumer sport applications. Sensors.

[19] Rana M., Mittal V. (2020). Wearable sensors for real-time kinematics analysis in sports: A review. IEEE Sens. J..

[20] Ahmadi A., Rowlands D., James D.A. (2009). Towards a wearable device for skill assessment and skill acquisition of a tennis player during the first serve. Sport. Eng..

[21] Delgado-García G., Vanrenterghem J., Ruiz-Malagón E.J., Molina-García P., Courel-Ibáñez J., Soto-Hermoso V.M. (2021). IMU gyroscopes are a valid alternative to 3D optical motion capture system for angular kinematics analysis in tennis. Proceedings of the Institution of Mechanical Engineers. Part P J. Sport. Eng. Technol..

[22] Pedro B., Cabral S., Veloso A.P. (2021). Concurrent validity of an inertial measurement system in tennis forehand drive. J. Biomech..

[23] Williams B.K., Sanders R.H., Ryu J.H., Bourdon P.C., Graham-Smith P., Sinclair P.J. (2019). Static and dynamic accuracy of a magnetic-inertial measurement unit used to provide racket swing kinematics. Sport. Biomech..

[24] De Luca C.J. (1997). The use of surface electromyography in biomechanics. J. Appl. Biomech..

[25] Moritz E.F., Haake S., Savage N., Subic A. (2006). Relating grip characteristics to the dynamic response of tennis racquets. The Engineering of Sport 6: Volume 2: Developments for Disciplines.

[26] Christensen J., Rasmussen J., Halkon B., Koike S. (2016). The Development of a Methodology to Determine the Relationship in Grip Size and Pressure to Racket Head Speed in a Tennis Forehand Stroke.

[27] Keaney E.M., Reid M. (2020). Quantifying hitting activity in tennis with racket sensors: New dawn or false dawn?. Sport. Biomech..

[28] United States Tennis Association (2004). Coaching Tennis Successfully.

[29] Morris M., Jobe F.W., Perry J., Pink M., Healy B.S. (1989). Electromyographic analysis of elbow function in tennis players. Am. J. Sport. Med..

[30] Elliott B., Reid M., Whiteside D. (2018). Biomechanics of Groundstrokes and Volleys. Tennis Medicine: A Complete Guide to Evaluation, Treatment, and Rehabilitation.

[31] Elliott B., Marsh T., Overheu P. (1989). A biomechanical comparison of the multisegment and single unit topspin forehand drives in tennis. J. Appl. Biomech..

[32] Knudson D.V., Blackwell J.R. (2005). Variability of impact kinematics and margin for error in the tennis forehand of advanced players. Sport. Eng..

[33] Bartlett R., Wheat J., Robins M. (2007). Is movement variability important for sports biomechanists?. Sport. Biomech..

[34] Preatoni E., Hamill J., Harrison A.J., Hayes K., Van Emmerik R.E., Wilson C., Rodano R. (2013). Movement variability and skills monitoring in sports. Sport. Biomech..

[35] Elliott B., Takahashi K., Noffal G. (1997). The influence of grip position on upper limb contributions to racket head velocity in a tennis forehand. J. Appl. Biomech..

[36] Rogowski I., Rouffet D., Lambalot F., Brosseau O., Hautier C. (2011). Trunk and upper limb muscle activation during flat and topspin forehand drives in young tennis players. J. Appl. Biomech..

[37] Genevois C., Reid M., Creveaux T., Rogowski I. (2020). Kinematic differences in upper limb joints between flat and topspin forehand drives in competitive male tennis players. Sport. Biomech..

[38] Takahashi K., Elliott B., Noffal G. (1996). The role of upper limb segment rotations in the development of spin in the tennis forehand. Aust. J. Sci. Med. Sport.

[39] Milner C.E. (2019). Functional Anatomy for Sport and Exercise: A Quick A-to-Z Reference.

[40] Genevois C., Amsallem C., Brandli C., Rogowski I. (2018). Using inertial sensors to monitor on-court tennis training sessions. Coach. Sport Sci. Rev..

[41] Rota S., Hautier C., Creveaux T., Champely S., Guillot A., Rogowski I. (2012). Relationship between muscle coordination and forehand drive velocity in tennis. J. Electromyogr. Kinesiol..

[42] Reid M., Elliott B., Crespo M. (2013). Mechanics and learning practices associated with the tennis forehand: A review. J. Sport. Sci. Med..

[43] Bollettieri N. (2001). Bollettieri’s Tennis Handbook.

[44] Knudson D., Elliott B. (2004). Biomechanics of tennis strokes. Biomedical Engineering Principles in Sports.

[45] Stuelcken M., Stuelcken M., Mellifont D., Gorman A., Sayers M. (2017). Wrist injuries in tennis players: A narrative review. Sport. Med..

[46] Gruchow H.W., Pelletier D. (1979). An epidemiologic study of tennis elbow: Incidence, recurrence, and effectiveness of prevention strategies. Am. J. Sport. Med..

[47] Nirschl R.P. (1974). The etiology and treatment of tennis elbow. J. Sport. Med..

[48] De Smedt T., de Jong A., Van Leemput W., Lieven D., Van Glabbeek F. (2007). Lateral epicondylitis in tennis: Update on aetiology, biomechanics and treatment. Br. J. Sport. Med..

[49] Pluim B.M., Windler G. (2018). Epidemiology of Tennis Injuries. Tennis Medicine: A Complete Guide to Evaluation, Treatment, and Rehabilitation.

[50] Scott A., Squier K., Alfredson H., Bahr R., Cook J.L., Coombes B., de Vos R.-J., Fu S.N., Grimaldi A., Lewis J.S. (2020). Icon 2019: International scientific tendinopathy symposium consensus: Clinical terminology. Br. J. Sport. Med..

[51] Ciccotti M.G., Ramani M.N. (2003). Medial epicondylitis. Sport. Med. Arthrosc. Rev..

[52] Alizadehkhaiyat O., Frostick S.P. (2015). Electromyographic assessment of forearm muscle function in tennis players with and without Lateral Epicondylitis. J. Electromyogr. Kinesiol..

[53] Shiri R., Viikari-Juntura E., Varonen H., Heliövaara M. (2006). Prevalence and determinants of lateral and medial epicondylitis: A population study. Am. J. Epidemiol..

[54] Roetert E.P., Brody H., Dillman C.J., Groppel J.L., Schultheis J.M. (1995). The biomechanics of tennis elbow: An integrated approach. Clin. Sport. Med..

[55] Li F.X., Fewtrell D., Jenkins M. (2004). String vibration dampers do not reduce racket frame vibration transfer to the forearm. J. Sport. Sci..

[56] Knudson D.V., White S.C. (1989). Forces on the hand in the tennis forehand drive: Application of force sensing resistors. J. Appl. Biomech..

[57] Hennig E.M.M., Thomas L. (1995). The Influence of Tennis Racket Characteristics and Grip Strength on the Magnitude of Arm Vibration in Tennis: Sports Medicine and Science 1995.

[58] Naß D., Hennig E.M., Schnabel G. Ball impact location on a tennis racket head and its influence on ball speed, arm shock and vibration. Proceedings of the 16 International Symposium on Biomechanics in Sports (1998).

[59] Naß D., Hennig E.M. (1998). The influence of impact location on the racket head on ball speed and load transfer to the arm during tennis serves. Proceedings of the Third North American Congress on Biomechanics.

[60] Hennig E.M. (2007). Influence of racket properties on injuries and performance in tennis. Exerc. Sport Sci. Rev..

[61] Hatze H. (1976). Forces and duration of impact, and grip tightness during the tennis stroke. Med. Sci. Sport..

[62] Wei S.-H., Chiang J.-Y., Shiang T.-Y., Chang H.-Y. (2006). Comparison of Shock Transmission and Forearm electromyography Between Experienced and Recreational Tennis Players During Backhand Strokes. Clin. J. Sport Med..

[63] Brody H., Cross R., Lindsey C. (2002). The Physics and Technology of Tennis.

[64] Pedro B., João F., Lara J.P., Cabral S., Carvalho J., Veloso A.P. (2022). Evaluation of upper limb joint contribution to racket head speed in elite tennis players using imu sensors: Comparison between the cross-court and inside-out attacking forehand drive. Sensors.

[65] Yeh I.-L., Elangovan N., Feczer R., Khosravani S., Mahnan A., Konczak J. (2019). Vibration-Damping technology in tennis racquets: Effects on vibration transfer to the arm, muscle fatigue and tennis performance. Sport. Med. Health Sci..

[66] Chadefaux D., Rao G., Le Carrou J.-L., Berton E., Vigouroux L. (2017). The effects of player grip on the dynamic behaviour of a tennis racket. J. Sport. Sci..

[67] Schnabel G., Hennig E. Wrist angular motion, grip strength and vibrational arm loads of casual and expert tennis players during forehand and backhand drives. Proceedings of the Ninth Biennial Conference of the Society of Biomechanics.

[68] Bulletin (2013). Real Low Latency Logging for Teensy 3.5/3.6 SDIO SD. https://forum.pjrc.com/threads/43834-Real-low-latency-logging-for-Teensy-3-5-3-6-SDIO-SD.

[69] Besomi M., Hodges P.W., Clancy E.A., Van Dieën J., Hug F., Lowery M., Merletti R., Søgaard K., Wrigley T., Besier T. (2020). Consensus for experimental design in electromyography (CEDE) project: Amplitude normalization matrix. J. Electromyogr. Kinesiol..

[70] McErlain-Naylor S.A. (2020). A Practical Open-Source Comparison of Discrete and Continuous Biomechanical Analysis Techniques. ISBS Proc. Arch..

[71] Bańkosz Z., Winiarski S. (2020). Statistical parametric mapping reveals subtle gender differences in angular movements in table tennis topspin backhand. Int. J. Environ. Res. Public Health.

[72] Pataky T.C. (2010). Generalized n-dimensional biomechanical field analysis using statistical parametric mapping. J. Biomech..

[73] Github Normality interpretation. spm1d 2017. https://github.com/0todd0000/spm1d/issues/77.

[74] Github The Interpretation of the Results spm1dmatlab 2016. https://github.com/0todd0000/spm1dmatlab/issues/29.

[75] Landlinger J., Lindinger S.J., Stöggl T., Wagner H., Müller E. (2010). Kinematic differences of elite and high-performance tennis players in the cross court and down the line forehand. Sport. Biomech..

[76] Konrad P. (2005). The abc of emg. A Pract. Introd. Kinesiol. Electromyogr..

[77] Rogowski I., Creveaux T., Triquigneaux S., Macé P., Gauthier F., Sevrez V. (2015). Tennis racket vibrations and shock transmission to the wrist during forehand drive. PLoS ONE.

[78] Chadefaux D., Rao G., Androuet P., Berton E., Vigouroux L. (2017). Active tuning of stroke-induced vibrations by tennis players. J. Sport. Sci..

[79] Rota S., Morel B., Saboul D., Rogowski I., Hautier C. (2014). Influence of fatigue on upper limb muscle activity and performance in tennis. J. Electromyogr. Kinesiol..

[80] De Monsabert B.G., Rossi J., Berton E., Vigouroux L. (2012). Quantification of hand and forearm muscle forces during a maximal power grip task. Med. Sci. Sport. Exerc..

[81] Github Minimum Number of Participants for Normality and t Tests spm1dmatlab 2021. https://github.com/0todd0000/spm1dmatlab/issues/154.

[82] Banwell G.H., Roberts J.R., Halkon B.J., Rothberg S.J., Mohr S. (2014). Understanding the dynamic behaviour of a tennis racket under play conditions. Exp. Mech..

